# Controlling the layer localization of gapless states in bilayer graphene with a gate voltage

**DOI:** 10.1088/2053-1583/aaa490

**Published:** 2018

**Authors:** W Jaskólski, M Pelc, Garnett W Bryant, Leonor Chico, A Ayuela

**Affiliations:** 1 Institute of Physics, Faculty of Physics, Astronomy and Informatics, Nicolaus Copernicus University, Grudziadzka 5, 87-100 Toruń, Poland; 2 Donostia International Physics Center (DIPC), Paseo Manuel Lardizabal 4, 20018 Donostia-San Sebastián, Spain; 3 Centro de Física de Materiales, CFM-MPC CSIC-UPV/EHU, Paseo Manuel Lardizabal 5, 20018 Donostia-San Sebastián, Spain; 4 Quantum Measurement Division and Joint Quantum Institute, National Institute of Standards and Technology, Gaithersburg, MD, 20899-8423, United States of America; 5 Instituto de Ciencia de Materiales de Madrid (ICMM), Consejo Superior de Investigaciones Científicas (CSIC), C/ Sor Juana Inés de la Cruz 3, 28049 Madrid, Spain; 6 Departamento de Física de Materiales, Facultad de Químicas, UPV-EHU, 20018 San Sebastián, Spain

**Keywords:** graphene, bilayer graphene, topologically protected states

## Abstract

Experiments in gated bilayer graphene with stacking domain walls present topological gapless states protected by no-valley mixing. Here we research these states under gate voltages using atomistic models, which allow us to elucidate their origin. We find that the gate potential controls the layer localization of the two states, which switches non-trivially between layers depending on the applied gate voltage magnitude. We also show how these bilayer gapless states arise from bands of single-layer graphene by analyzing the formation of carbon bonds between layers. Based on this analysis we provide a model Hamiltonian with analytical solutions, which explains the layer localization as a function of the ratio between the applied potential and interlayer hopping. Our results open a route for the manipulation of gapless states in electronic devices, analogous to the proposed writing and reading memories in topological insulators.

## 1. Introduction

Two-dimensional Dirac materials, like graphene, have attracted remarkable interest for novel nanoelectronic applications due to their reduced dimensionality and extraordinary transport properties [1–6]. Recently, great effort has been devoted to seek and develop methods to control the transport of different degrees of freedom in these new materials. Spin [7], valley [8, 9], angular momentum [10] or cone [11] are being proposed in addition to charge, as a means to convey and store information in future devices. In this field, bilayer graphene (BLG) stands out as a promising candidate for nanoelectronics [12–16]. In its most common form, the so-called Bernal or AB-stacked BLG, an energy gap can be opened and tuned by an applied gate voltage [17–20], which is not possible in single-layer graphene.

**Figure 1.**
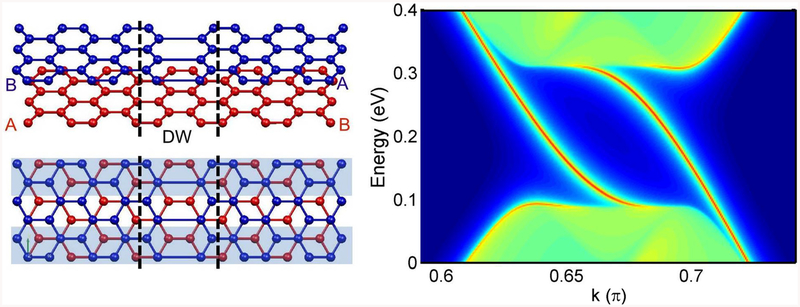
Left: Geometry of a minimal stacking domain wall in bilayer graphene, separating regions with AB ad BA stacking. Right: Topological modes arising at the gap in gated bilayer graphene with a stacking domain wall. Lattice constant *a* = 1.

The gap in AB-stacked BLG arises because of the combination of two factors: the interlayer hopping permits to distinguish A and B atoms, breaking sublattice symmetry, and the gate potential breaks inversion symmetry, differentiating the two layers (figure 1 left). Importantly, when a domain wall (DW) divides bilayer graphene into AB and BA stacking domains, a pair of states appears at each valley, connecting the valence and conduction band continua through the energy gap (figure 1 right) [21–23]. These gapless states are topologically protected if the valley index is conserved. Recent experiments [24, 25] show the gapless states localized along the DW [25], being robust conducting channels [22–24, 26].

Gapless states in bilayer graphene have also been studied theoretically in works focusing on their topological features [27–29]. As long as the no-valley mixing condition is fulfilled, one can specify a topological quantity, namely, the valley Chern number [27, 29]. Its change across the DWs accounts for the number of gapless states that appear under a gate potential. However, experiments and applications based on these conducting states need a clarification of other important physical features, such as spatial localization, where using topology arguments would be complex. A microscopic model is better suited to identify the symmetry and localization of the states. A few relevant works on lattice models specifically study different stacking boundaries for a gate voltage close to the interlayer coupling [27, 28]. However, a systematic analysis of the layer localization in these stacking DW states under varying gate voltages is still missing.

In this work we show that the layer localization of gapless states in a gated BLG domain wall is tuned by an externally applied voltage. We demonstrate that at the domain wall the carriers are concentrated in the upper or bottom layer depending on the ratio between gate voltage and interlayer coupling. This dependence allows for switching the localization of topological states between layers by a change of the gate magnitude, implying that an additional degree of freedom, the layer, can play a role in BLG-based devices. Next, to explain the aforementioned behavior, we consider the symmetries of the bands and bonds formed between atoms in the gated system. We model a periodic array of stacking domain walls that, in contrast to the single DW, allows us to work with energy bands and wave functions. In this way, we identify the specific bands of the uncoupled layers which create the gapless states under interlayer coupling.

Controlling the layer localization by an external voltage opens a novel route for the manipulation of gapless states in electronic devices that could be denominated *layertronics*, in analogy to valley- and spintronics. We propose that layer localization would be another tunable degree of freedom in BLG, in addition to valley and spin.

## 2. Gated stacking domain wall: preliminary remarks

We first review the main features of the system. The left panel of figure 1 shows a single domain wall between AB and BA regions in bilayer graphene joined in the zigzag direction. Strained or corrugated graphene presents larger and more realistic domain walls, but for our theoretical analysis we choose an abrupt boundary that allows for an AB/BA stacking change. Previous works [23, 24, 28, 30] have shown the robustness of topological states for smoother boundaries, with their main features preserved. For such a single boundary under a gate there are two topological gapless modes around the *K* point, as shown in the right panel of figure 1. The plot shows the local density of states (LDOS) resolved in energy *E* and wave vector *k*. These calculations employ a Green’s function matching method^7^ and a *p*_*z*_ tight-binding model, with an intralayer hopping parameter *γ*_0_ = −2.7 eV and a single interlayer hopping *γ*_1_ = 0.1*γ*_0_ [12, 13]. There is another valley with another couple of gapless states with negative wave vectors with respect to figure 1 due to time-reversal symmetry. Note that the valley separation has motivated the proposal to employ such topologically protected modes for graphene valleytronics [31, 32].

## 3. Layer localization with gate voltage magnitude

**Figure 2.**
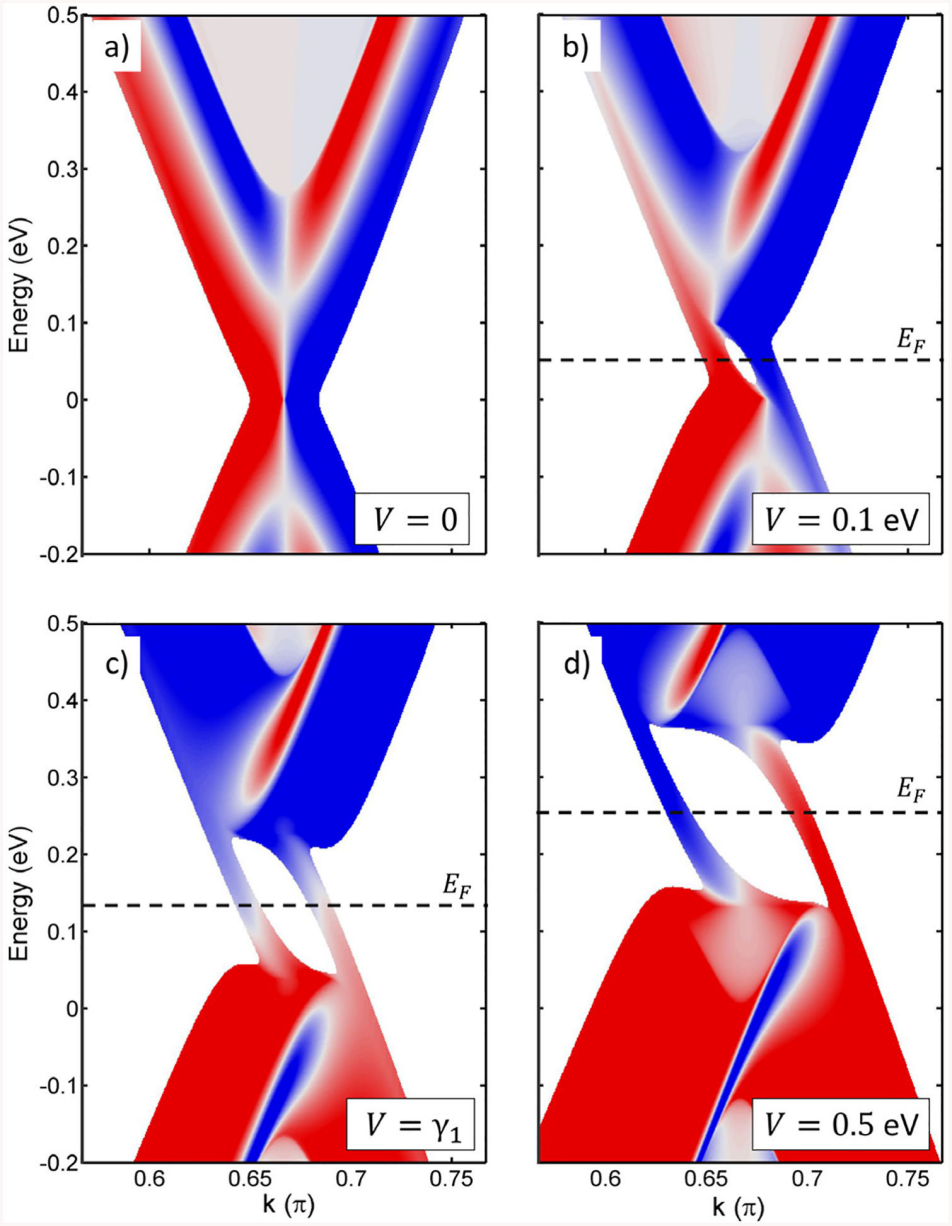
Topological modes of bilayer graphene with a stacking domain wall: ungated (a) and when the gate voltage is applied to the bottom layer: *V* = 0.1 eV (b), *V* = *γ*_1_ (c) and *V* = 0.5 eV (d). Color scale reflects the localization in top (blue) or bottom (red) layer. Dashed lines indicate the Fermi levels. For *V* = 0 the Fermi level is at zero-energy, while for *V* > 0—in the middle of the gap, at *E* = *V*/2.

We report on the effect of layer localization exchange of topological states at a single stacking domain wall for small and large gate voltages. We use the same Green’s function matching method and graphene model when there is an applied *V*. The LDOS around the *K* valley projected in the boundary nodes is shown in figure 2 for different voltages. The localization in the top and bottom layers is presented by the color scale from blue to red^8^. We start with the LDOS for ungated bilayer in panel (a). The LDOS localizes differently at the top and bottom layers because the symmetry between them is broken by the stacking domain wall. For a small gate voltage applied to the bottom layer, e.g. *V* = 0.1 eV in panel (b), the gap states appear, as it is well known, but they turn out to be separated in the two layers. The state on the left side of the cone is more localized at the bottom layer, while the state on the right is at the top layer. This localization can be explained as a perturbation of the LDOS of the ungated bilayer for small voltages applied. For increasing voltages, around *V* = *γ*_1_ in panel (c), the states become fully mixed between layers. Next, for a large voltage, e.g. *V* = 0.5 eV in panel (d), the states are again separated in the top and bottom layer. This time, however, the state on the left side of the cone is more localized at the top layer, while the state on the right is at the bottom layer. Therefore, the topological states at the boundary atoms reverse localization for small and large voltages of the same sign.

**Figure 3.**
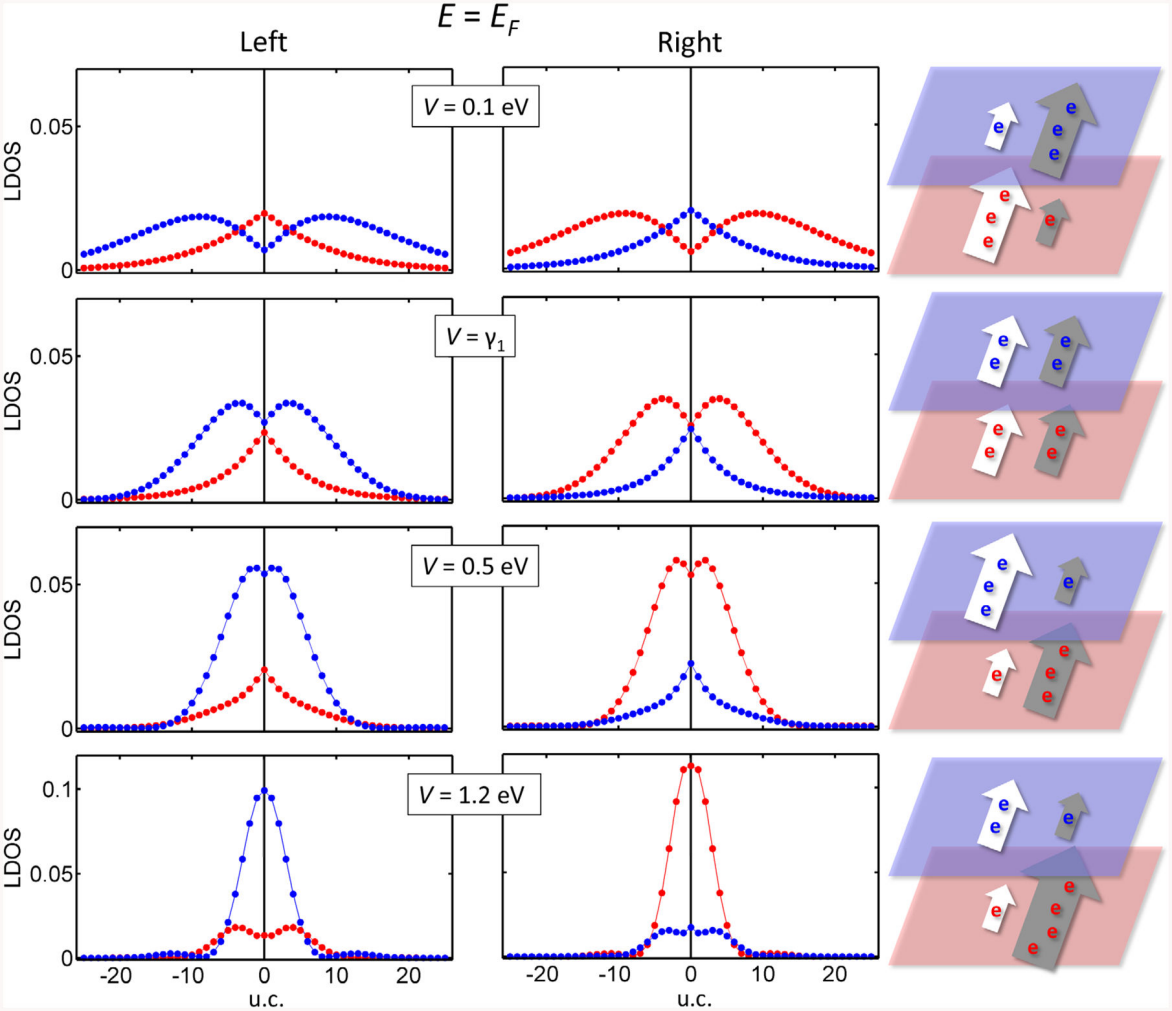
Unit-cell averaged LDOS at the bottom (red) and the top layer (blue) plotted for both topologically protected states at the Fermi level for different gate voltages. The horizontal axis indicates the distance (in unit cells) from the boundary (DW). For each case we include schemes putting both states side by side—left and right topological states are presented by white and grey arrows, respectively. The sizes of the arrows and the number of electrons reflect the quantitative values of the localized LDOS per layer for each state. For high voltage the left and right topological states are not complementary, summing up to a large contribution to the bottom layer.

Concerning the maximum experimental voltage between layers we have to take into account the maximum field applied by gate materials used in experiments. For instance, the breakdown field in standard SiO_2_ substrates would allow a bias in bilayer graphene under dual gating up to 0.6–0.7 eV. These biases are larger than the 0.5 eV bias for which the layer switching is clearly seen, as discussed above in figure 3. As a limiting voltage and for the sake of completeness, we show in figure 3 the case for *V* = 1.2 eV bias, that allows us to comment on the asymmetry of the cones around specific *K* and *K*′ points.

One can also ponder the role of screening on layer localization under such voltages. It is noteworthy that DFT calculations [33], which take into account such effects, show that electron Coulomb screening between layers results in a smaller interlayer coupling. We have performed calculations for other smaller *γ*_1_ values keeping either the *γ*_0_ value or the *γ*_0_/*γ*_1_ ratio fixed, as reported in DFT calculations. Including further electron screening by reducing the interlayer hopping *γ*_1_ would decrease the required gate potentials for top/bottom layer polarization to smaller values. The layer polarization with gate value is preserved even when modifying the screening between layers to more realistic values (See suppl. inf. (stacks.iop.org/TDM/5/025006/mmedia), SI1, figure S1 for details). Additionally, note that the inclusion of an on-site Coulomb term in our model amounts to a higher localization of the electron. We have checked that it does not change our results for reasonable values of *U* around *γ*_0_ [34, 35].

### 3.1. Spatial distribution of TPS in each layer

Now we explore how the localization of topological states extends away from the boundary atoms. We investigate their spatial distributions at the Fermi level, as shown in figure 3. The LDOS of each state is averaged per rectangular zigzag unit cell (four atoms in each layer) and decomposed in the top and bottom layers. The spatial distributions show that localized states have their maxima close to the boundary region and decay in the adjacent unit cells [23]. The weight of the LDOS at the top and bottom layers is interchanged by increasing the voltage, around *V* ≈ *γ*_1_, as commented above.

It is also important to compare with the experiment to consider how large the region of layer interchange extends. Because of their gapless character, the states decay away from the domain wall in AB and BA stacking regions. Their decay rates increase with magnitude of the gate voltage. Note that the region where a state exists with a given layer polarization can be limited by its oscillatory character or fast decay (see cases *V* = 0.1 eV and *V* = 1.2 eV in figure 3). In an abrupt domain wall, the width of the top/bottom layer polarization extends at least for 6 u.c., i.e. about 4 nm. In the case of a smooth domain wall, such as induced by a tensile, shear or corrugated layer, we can expect two effects. On one hand, the finite transition region may affect the exact spatial distribution of the states and the gate voltage needed for the polarization change may slightly decrease. On the other hand, the interchange region will be extended with respect to the case for a sharp domain wall. Because the transition region acts as a gapless medium [23, 28, 36], TPS will propagate therein as extended states preserving their high or low localization in each layer [23], and showing the top/bottom layer localization even better resolved.

The layer localization of the gapless states with gate voltage exists also in the case of a smooth domain walls. The gate voltage for which we observe the change of the top/bottom polarization can slightly decrease, e.g. for the case of corrugated domain walls. Note that when the domain wall region is smooth, the interchange region increases. The major finding on top/bottom layer localization of TPS with gate value remains the same when the domain walls are smooth.

**Figure 4.**
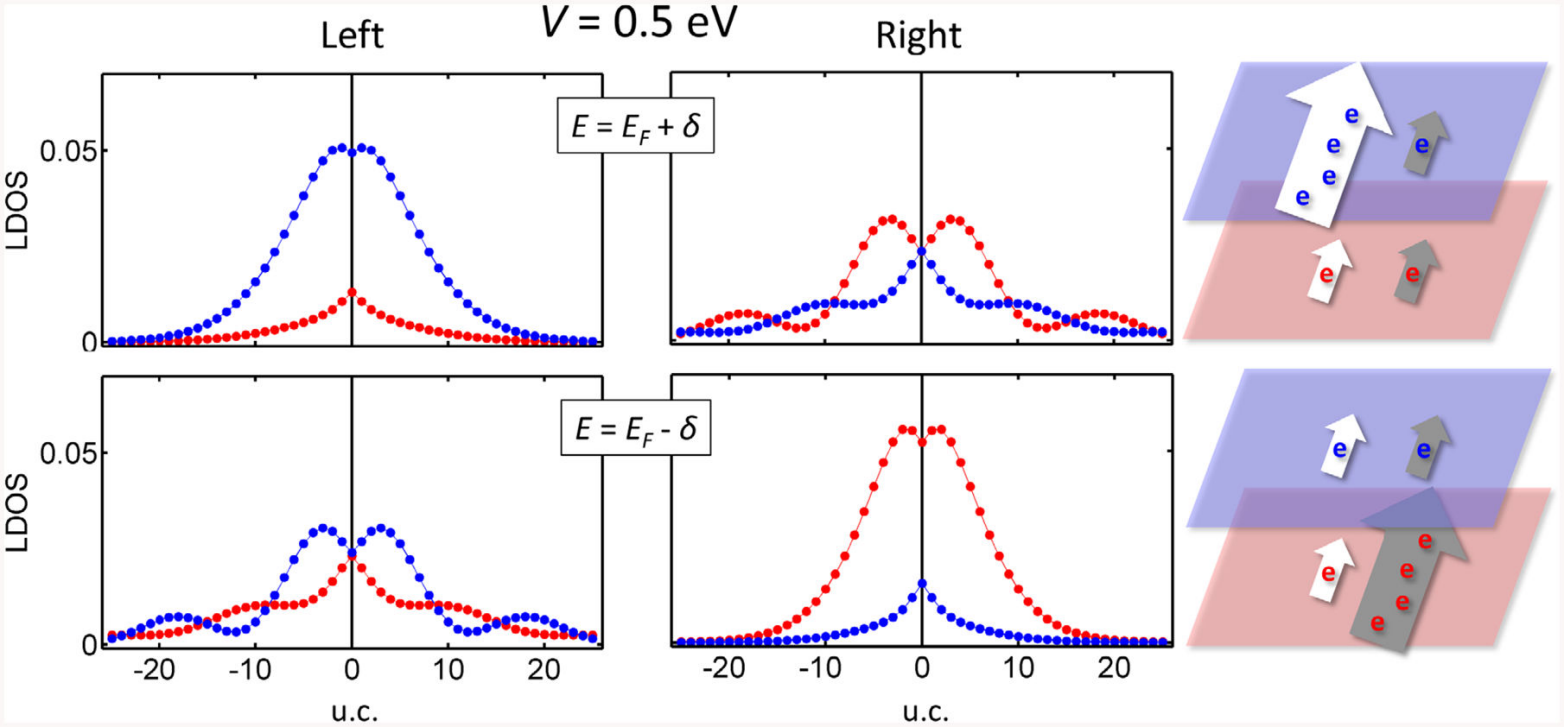
Unit-cell averaged LDOS at the bottom (red) and the top layer (blue) plotted for both topologically protected states for *V* = 0.5 eV above and below the Fermi level; *δ* = 0.1 eV. Schemes of the two topological states follow the notation given in figure 3. The layer localization is now distinct at voltages accessible in experiments.

Topological states in gated bilayer graphene run across the insulator gap, so that their behavior can also be investigated by varying the chemical potential. Note that up to now we have looked at the topological states at the Fermi energy. However, experiments are usually performed away from the neutrality point, due to doping or to the interaction with different substrates. Figure 4 shows the LDOS distributions of topological states above and below the Fermi level. The left and right topological states are no longer complementary. We find that the total charge is more localized on one of the layers, i.e. layer localization is tuned by doping. This effect seems similar to the above case with high voltages, but with significant differences because: (i) the topological states can be on either top or bottom layer, and (ii) the effect is available at lower gate voltage values. Additionally, the distribution of the topological states in the top or bottom layers can be controlled by the experimental gate voltage applied in doped samples, a finding that has to be taken into account for practical applications.

As far as we know, this effect based on the asymmetry in localization of topological states has not been noticed and not explored before. We believe that the reason for this omission is that for *V* ≈ *γ*_1_, as normally set in most calculations, the energy gap saturates at that value and the topological states are equally disrtibuted between the two layers [23, 28, 37]. The layer distribution for these potential values is fully mixed at the boundary atoms.

## 4. Insight into topological gapless states

### 4.1. Periodic stacking domain walls

To gain physical insight into the remarkable variation of the spatial distribution of these modes, we need to examine the symmetry of the corresponding wave functions. To this end, we study periodic systems, i.e. bilayer superlattices of stacking domain walls along the zigzag direction [38, 39], which we label BS-DW^9^. The unit cell and band structures near the K point for different superlattice lengths are collected in figure S2 in supplementary information. It is important to note that for *V* > 0 and *γ*_1_ = 0 the bands of the constituent graphene layers overlap. For nonzero *γ*_1_ the overlapping bands interact and split yielding the energy gap in the case of pristine bilayer. However, when a stacking domain wall is imposed, two bands still persist in the gap. The treatment of *γ*_1_ as a perturbation allows recognizing the bands of single graphene layers that give rise to the presence of topological bands in the energy gap, to be analyzed below.

### 4.2. Gaps and band crossing points near the Fermi level

**Figure 5.**
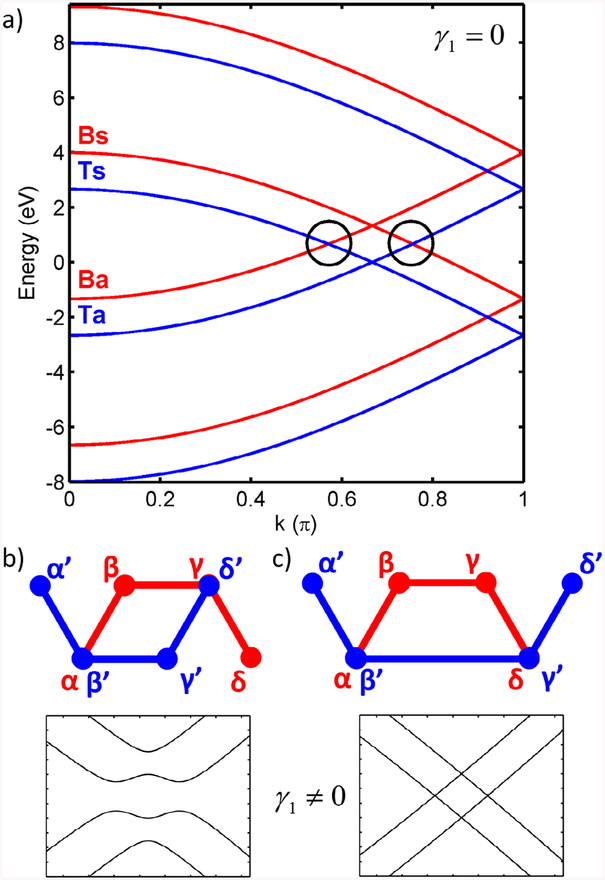
(a) Bands of two uncoupled layers of graphene, with a gate voltage applied to the bottom layer, for an 8-atom rectangular unit cell, shown below. Red and blue bands correspond to bottom and top layers, respectively. Bands are labeled according to localization and symmetry; circles mark the crossing points analyzed below, see text. Rectangular 8-atom unit cells, with all atoms labeled, for (b) AB bilayer graphene and (c) minimal double stacking domain wall, i.e. a BS-DW with *W* = 1. The resulting band structures close to the crossing points are shown below the respective unit cells. Greek symbols in (b) and (c) enumerate the nodes in the 8-atom unit cell.

We consider two uncoupled graphene layers with a gate potential *V* applied to the bottom layer and we switch on the interlayer hopping to study its role in the appearance of the topologically protected bands. Without hopping, the energy structure of two pristine graphene layers with a voltage difference applied is gapless, as shown in figure 5(a). We have chosen an 8-atom unit cell to compare more easily to the band structures of stacking DW superlattices. The bands of the gated bottom layer are shifted in energy by an amount *V* with respect to the top layer bands. At *k* = 2/3*π* the pair that belongs to the top ungated layer crosses at *E* = 0, while the pair of bands of the bottom gated layer cross at *E* = *V*. Note that the bands originating from different layers cross at $E=\frac{1}{2}V$. One pair crosses for *k* ≲ 2/3*π* and another pair for *k* ≳ 2/3*π*. The bands are labeled *B*, *T*, indicating that they belong to the bottom and top layers, respectively, and *s*, *a*, due to the symmetric or antisymmetric character of the corresponding wavefunctions.

When the interlayer interaction is switched on in the BLG case (figure 5(b)), the crossing bands mix and split because they form pairs of bonding and antibonding states and a gap opens. The resulting band structure near the Fermi level has the well-known Mexican hat shape (see the bands plotted in figure 5(b) below the unit cell) [12]. However, when the nodes are connected producing the stacking defects, as in figure 5(c), a pair of states remains in the gap, shown in the band structure depicted under the corresponding unit cell. The resulting structure is a BS-DW with *W* = 1. A key question that we address below is why these states are gapless and how they arise from the bands of pristine graphene.

**Figure 6.**
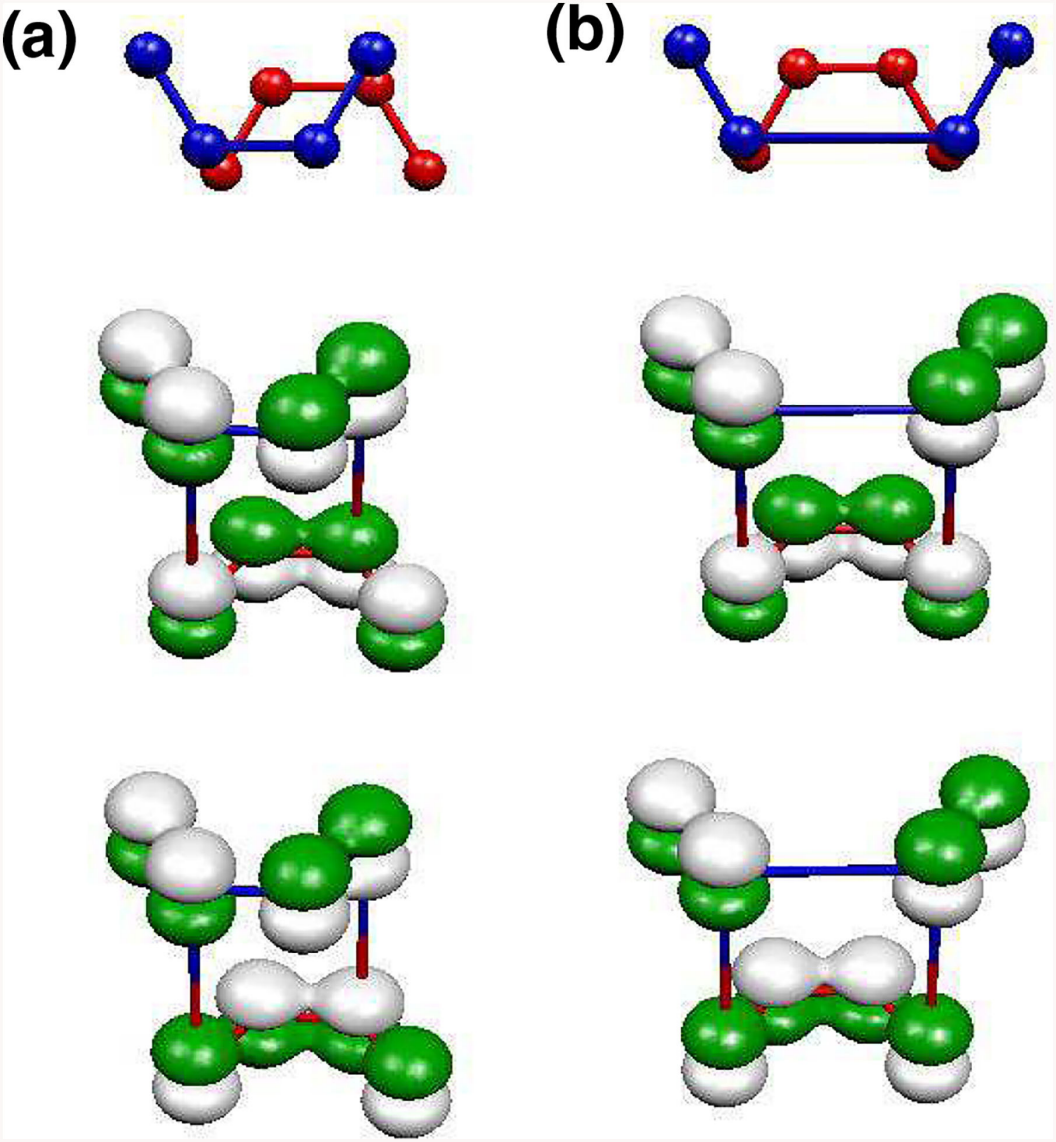
Scheme of the interlayer bonds between *p*_*z*_ orbitals at the left crossing point (see figure 5(a)), for (a) bilayer graphene with an 8-atom unit cell and (b) a BS-DW of width *W* = 1. Top and bottom layers are colored in blue and red, respectively. The bonding and antibonding orbitals between the *Ts* and *Ba* wavefunctions are shown below. Green and white colors denote the sign of *p*_*z*_ lobes. The vertical blue/red lines show the atoms in the top layer that couple to atoms in the bottom layer. For pristine bilayer graphene, the combinations of the *p*_*z*_ orbitals yield different bonding and antibonding solutions, Ψ_±_. No such solutions can be formed in the BS-DW case.

We focus on the left crossing point marked with a circle in figure 5(a). The two bands crossing therein are labeled *Ts* and *Ba*, due to their symmetry and localization. The bonding and antibonding *p*_*z*_ orbital combinations formed due to the interlayer interaction are schematically illustrated in figure 6(a). However, for a domain wall, there are always topological modes crossing the gap. Figure 6(b) graphically shows that it happens because it is not possible to form bonding and antibonding combinations of the top and bottom layer wave functions simultaneously for both pairs of the overlapping nodes from different layers in a DW.

### 4.3. Basis functions for the crossing bands

We next treat the interlayer hopping as a perturbation to the bands of the uncoupled layers. The starting basis is given in terms of the uncoupled bands depicted in figure 5(a). For the 8-atom unit cell employed therein, and explicitly labeled in figure 5(b), we have

(1) $$\Psi_{Ba,k}=\frac{1}{2}\left(\phi_{\alpha }+{\text{e}}^{\text{i}kd}\phi_{\beta }-{\text{e}}^{\text{i}kd}\phi_{\gamma }-\phi_{\delta }\right)\;\Psi_{Bs,k}=\frac{1}{2}\left(\phi_{\alpha }-{\text{e}}^{\text{i}kd}\phi_{\beta }-{\text{e}}^{\text{i}kd}\phi_{\gamma }+\phi_{\delta }\right)\;\Psi_{Ta,k}=\frac{1}{2}\left({\text{e}}^{\text{i}kd}\phi_{\alpha^{\prime }}+\phi_{\beta^{\prime }}-\phi_{\gamma^{\prime }}-{\text{e}}^{\text{i}kd}\phi_{\delta^{\prime }}\right)\;\Psi_{Ts,k}=\frac{1}{2}\left({\text{e}}^{\text{i}kd}\phi_{\alpha^{\prime }}-\phi_{\beta^{\prime }}-\phi_{\gamma^{\prime }}+{\text{e}}^{\text{i}kd}\phi_{\delta^{\prime }}\right)$$

where the labels refer to their layer localization and their symmetry, as in figure 5(a); *d* is the distance between contiguous rows of atoms in the zigzag direction (see figure 6); *ϕ*_*μ*_ denotes the *p*_*z*_ orbital in the *μ* atom, with *μ* running from *α* to *δ* in the bottom layer and the same labels with primes in the top layer (see figures 5(b) and (c)). The wave vector *k* dependence is explicitly indicated as a subscript, but we omit it from now on for the sake of simplicity.

For connected layers, the degeneracy at the crossing points of the uncoupled system $\left(k\sim \frac{2}{3}\pi \right)$, marked in figure 5(a) with circles, can be lifted. Focusing in the band wavefunctions at the left crossing point, we construct the bonding and antibonding combinations $\Psi_{+}=\frac{1}{\sqrt{2}}\left(\Psi_{Ts}+\Psi_{Ba}\right)$ and $\Psi_{-}=\frac{1}{\sqrt{2}}\left(\Psi_{Ts}-\Psi_{Ba}\right)$, respectively. When the interlayer coupling is switched on, these states are shifted in energy by an amount given by 〈Ψ_±_|*H*^TB^|Ψ_±_〉, where *H*^TB^ is the interlayer coupling Hamiltonian.

By connecting the layers, pristine gated bilayer graphene is obtained; to this purpose the connected atom pairs are *α* − *β*′ and *γ* − *δ*′, see figure 5(b). The energy shift of the bonding state from the energy at the crossing point is given by

$$\langle \Psi_{+}\left|H_{\text{BLG}}^{\text{TB}}\right|\Psi_{+}\rangle =-\frac{1}{2}\left|\gamma_{1}\right|.$$

Analogously, the energy shift of the antibonding state is

$$\langle \Psi_{-}\left|H_{\text{BLG}}^{\text{TB}}\right|\Psi_{-}\rangle =\frac{1}{2}\left|\gamma_{1}\right|,$$

so that the total gap equals |*γ*_1_|. This is why the bands *Ts* and *Ba* split at the left crossing point of figure 5(a). At the right crossing point it happens the same, but now for the *Ta* and *Bs* bands.

Along the same line of reasoning, we discuss why two pairs of the overlapping bands survive in a periodic gated domain wall. The unit cell is shown in figure 5(c). In the previous case the two pairs of the overlapping nodes from the red and blue layers were different. At the left crossing point, the two Ψ_*Ts*_ ± Ψ_*Ba*_ functions yield a bonding combination of the *p*_*z*_ orbitals at one pair of the connected nodes, but an antibonding combination at another pair of nodes, see figure 6. In other words, the interlayer Hamiltonian changes, because now the connected nodes are *α* − *β*′ and *δ* − *γ*′, as depicted in figure 5(b). With this different coupling,

$$\langle \Psi_{\pm }\left|H_{\text{DW}}^{\text{TB}}\right|\Psi_{\pm }\rangle =0,$$

and the bands *Ts* and *Ba* still have a crossing point. A similar analysis can be performed for the right crossing point for the *Ta* and *Bs* bands. As a result, we end up with two pairs of states connecting the valence and conduction band, i.e. the topological modes.

**Figure 7.**
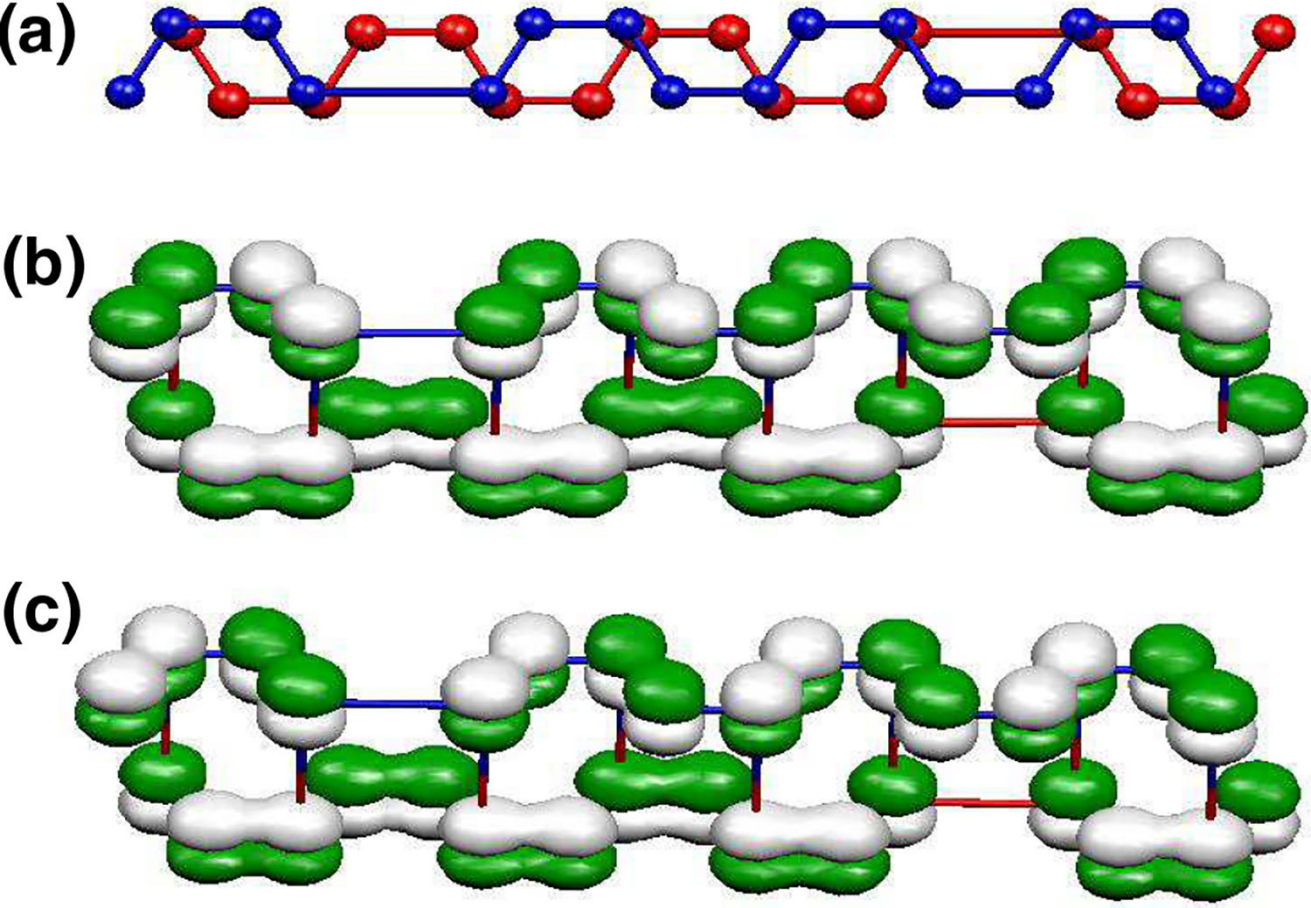
Geometry (a) and distribution of the signs of coefficients of the wave functions Ψ_+_ (b) and Ψ_−_ (c) in the unit cell of BS-DW with *W* = 4.

This reasoning can be applied to a BS-DW of arbitrary width. In such a case, one can choose a bilayer graphene superlattice of the same width *W* = *n*, consider the uncoupled layer case, which yields a band structure similar to figure 7(a) but with 4*n* bands. In such instance, the bands crossing at *E*_F_, i.e. the 2*n* and 2*n* + 1 bands have exactly the same antisymmetric and symmetric character as those at the crossing points analyzed for the 8-atom case. The difference in the wavefunctions is the normalization factor, being $\frac{1}{\sqrt{N}}$, with *N* = 4*n* is the number of atoms in the unit cell. When the coupling is switched on and the geometry corresponds to a BS-DW, the energy shifts corresponding to Ψ_+_ and Ψ_−_ are zero, so there are two pairs of bands crossing the gap. Figures 7(b) and (c) illustrates the Ψ_±_ band wave functions for the case of *W* = 4. The sign of the *p*_*z*_ orbitals at the domain walls are exactly as those found for the minimal BS-DW.

**Figure 8.**
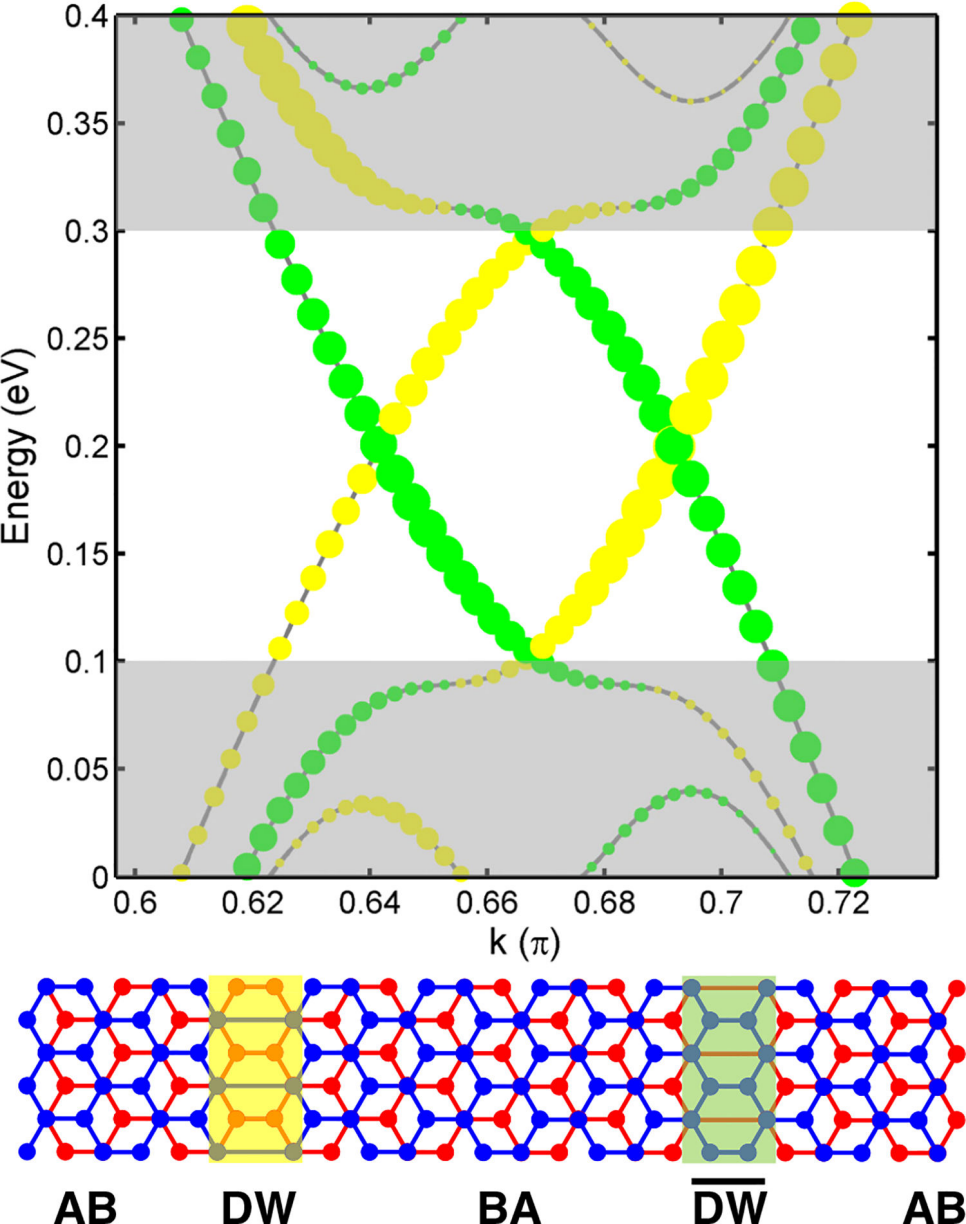
Bands of BS-DW of width *W* = 40 at gate voltage *V* = 0.4 eV resolved into the two stacking domain walls present in unit cell: DW—yellow and $\bar{\text{DW}}$—green, marked also on the scheme below. The size of the dots is related to the square of the module of wavefunction on the particular stacking domain wall nodes. Shaded areas mark the valence and conduction band continua for the single DW case. Four localized gap bands are in the non-shaded area.

**Figure 9.**
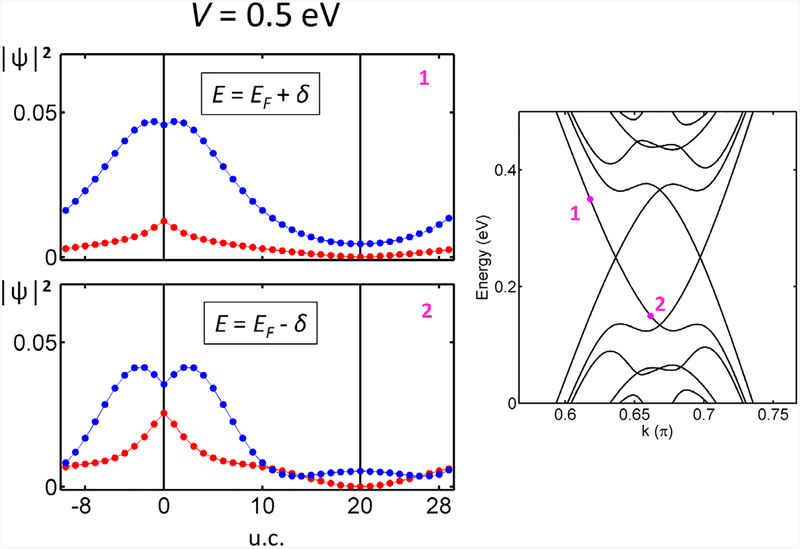
Unit-cell averaged Ψ*Ψ of BS-DW of width *W* = 40 at the bottom (red) and the top layer (blue) plotted for one of the topologically protected states for *V* = 0.5 eV above and below the Fermi level; *δ* = 0.1 eV. The difference in localization is similar to a single domain wall in figure 4.

As commented before, in a BS-DW one pair of the bands crossing the gap has positive velocity and the other pair has a negative slope. In the smallest BS-DW with *W* = 1, the states at the boundaries are unavoidably mixed. For large *W*, each pair belongs to a different stacking domain wall in the unit cell, being spatially separated, as illustrated in figure 8. The bands for *W* = 40 are resolved in the two stacking boundaries, DW and $\bar{\text{DW}}$. When one stacking DW is present in the system, one pair of bands is in the gap with the same velocity, as shown in figure 1. The sign of the velocity is related to the change of stacking, either AB to BA or vice versa. Further, the different localization between layers predicted from the periodic calculations are compared with the case for a single DW, as shown in section 3. In figure 9 we present the wavefunctions corresponding to *E*_*f*_ ± *δ* around the DW. We find that a DW either isolated or in a periodic arrangement behaves the same way.

### 4.4. Continuum Hamiltonian for topological states

We generalize the above discussion to *k* values away from the band crossing points. The Hamiltonian *H* of each layer close to the Dirac point is represented in the basis (1), given by Ψ_*Ba*_ and Ψ_*Bs*_ for one layer, and Ψ_*Ta*_ and Ψ_*Ts*_ for the other layer. For a monolayer, *H* is a 2 × 2 diagonal matrix with linear *k*, and −*k* values in the diagonal elements. For gated bilayer graphene we have to double this matrix [2, 37]. Using the basis{Ψ_*Ba*_, Ψ_*Bs*_, Ψ_*Ta*_, Ψ_*Ts*_} we obtain the interaction Hamiltonian between layers that introduces the hopping *γ*_1_ in the non-diagonal elements. Employing the expression of these basis vectors in terms of the localized orbitals *ϕ*_*μ*_ given in equation (1) and the geometry of the interlayer bonds shown in figure 5(b), the Hamiltonian for bilayer graphene is written

(2) $$H_{\text{BS}}=\left(\begin{array}{cccc} \alpha k+V & 0 & \frac{1}{2}\gamma_{1} & -\frac{1}{2}\gamma_{1}\\ 0 & -\alpha k+V & \frac{1}{2}\gamma_{1} & -\frac{1}{2}\gamma_{1}\\ \frac{1}{2}\gamma_{1} & \frac{1}{2}\gamma_{1} & \alpha k & 0\\ -\frac{1}{2}\gamma_{1} & -\frac{1}{2}\gamma_{1} & 0 & -\alpha k \end{array}\right),$$

where $\alpha =\sqrt{3}a\gamma_{0}/2\hbar$, and *a* is the graphene lattice constant. For *V* = 0 the eigenvalues of this Hamiltonian have a quadratic dependence on *k*, as it should be in bilayer graphene [37]. Two of them are degenerate at the Dirac point. For *V* ≠ 0 the gap opens and the low energy bands versus *k* show the Mexican hat shape. In the gated system the energy gap *E*_*g*_ depends on the parameters *V* and *γ*_1_. For *V* < *γ*_1_, *E*_*g*_ ≈ *V*, while for *γ*_1_ < *V*, *E*_*g*_ ≈ *γ*_1_. The Hamiltonian (2) gives an appropriate description of the low-energy bands of BLG.

In the same way, i.e. using the basis vectors given in (1), we derive a Hamiltonian for bilayer graphene with periodic stacking domain walls as,

(3) $$H_{\text{BS}-\text{DW}}=\left(\begin{array}{cccc} \alpha k+V & 0 & \frac{1}{2}\gamma_{1} & 0\\ 0 & -\alpha k+V & 0 & -\frac{1}{2}\gamma_{1}\\ \frac{1}{2}\gamma_{1} & 0 & \alpha k & 0\\ 0 & -\frac{1}{2}\gamma_{1} & 0 & -\alpha k \end{array}\right).$$

The Hamiltonian (3) includes a *k* dependence, but it does not mix *k* and −*k* values inside or between the layers. The eigenvalues remain linear in *k*, so for *V* ≠ 0 they cross the energy gap constituting the topologically protected gap states. Its eigenvalues have the following analytical expression:

(4) $$E=\pm \alpha k+V/2\pm \frac{1}{2}\sqrt{\gamma_{1}^{2}+V^{2}}.$$

The corresponding eigenvector components for velocity +*k* are

(5) $$\left(0,\frac{-V\pm \sqrt{\gamma_{1}^{2}+V^{2}}}{\gamma_{1}},0,1\right)$$

and for velocity −*k*

(6) $$\left(\frac{V\pm \sqrt{\gamma_{1}^{2}+V^{2}}}{\gamma_{1}},0,1,0\right).$$

Note that the localization of the states will behave differently in the two gate voltage regimes. In the limit of *V* ≪ *γ*_1_ the eigenvectors are mixed between layers—in contrast to the single domain wall case, where we observe localization reversal at the boundary. The reason for layer separation was the asymmetry between layers, which is no longer present in BS-DW.

In the limit of *V* ≫ *γ*_1_ the components of the eigenvectors given by equation (5) approach (0, 0, 0, 1) and those given by equation (6) approach (0, *V*/*γ*_1_, 0, 1), and to (0, 0, 1, 0) and (*V*/*γ*_1_, 0, 1, 0). This means that each pair of the gap states with the same velocity (+*k* or −*k*) has the corresponding wave functions localized in different layers. The wave functions in each pair have the same symmetry, i.e. *a* or *s* (see equation (1)). Therefore, the topologically protected gap states look like the single layer bands that cross at the Fermi level. This continuum Hamiltonian with four bands allows us to predict the relevant properties of topological states in stacking domain walls.

## 5. Discussion and conclusions

We have investigated the gapless states with topological character that appear in gated bilayer graphene with stacking domain walls. By employing atomistic models, we find that each of the two topological states in a valley is layer-resolved; furthermore, their localization is switched between the top and bottom layer by varying the magnitude, but not the sign, of the gate voltage. Therefore, besides the valley and sublattice degrees of freedom, these states can also be labeled by a layer index.

Our findings also bring forth an atomistic understanding of the origin of gapless states with topological character. We have analyzed the chemical bond formation between gated layers of graphene with domain walls to elucidate the appearance of gapless states. We have also provided a continuum model for the gapless states that correctly describes the swithing in layer localization of the topologically protected states. For a large gate voltage, i.e. above the interlayer coupling, the layer localization presents the standard trend between top and bottom layer for a particular voltage orientation. However, for a small gate voltage, the asymmetry between layers introduced by the stacking domain walls prevails, so the gapless states are slightly perturbed by this voltage, having an opposite layer localization.

Furthermore, we have shown that the layer spatial distribution of the topologically protected states is modified by doping and tuned by the gate voltage. The layer LDOS can be directly measured with an STM; this tool can also allow for further engineering on topologically protected states by adsorbing and moving molecules along the stacking domain walls. Controlling the carriers localization in distinct layers along domain walls would open the possibility for the design of *layertronic* devices, which could be exploited in addition to other degrees of freedom, like valley, spin and charge, in graphene-based electronics.

Any TPS in other materials could show similar layer localization control with the gate voltage, at least if there are two layers and two states involved, although the asymmetry induced by domain walls seems also required. The focus here has been on graphene-based materials. The hope is that this will stimulate interest in other materials that could also display layertronics.

## Supplementary Material

2

## Footnotes

7 The LDOS is calculated at the boundary in the zigzag direction. For details see [38, 39, 40, 41].

8 The LDOS ratio is plotted only when LDOS is greater than some set value.

9 The choice of periodic systems allows us to avoid the interplay between topological states and edge states characteristic for zigzag graphene edge [42].
