# Supplementary material for: Controlling the layer localization of gapless states in bilayer graphene with a gate voltage
Source: 2d Mater. Author manuscript; Available in PMC 2020 Feb 28. (PMC7047727; doi:10.1088/2053-1583/aaa490)
Supplement: 2 [file NIHMS1541656-supplement-2.pdf]

Supplementary Information for "Controlling the layer localization of gapless states in bilayer graphene with a gate voltage"

W. Jaskólski, M. Pelc, Garnett W. Bryant, Leonor Chico, and A. Ayuela

SI1. LOWERED INTERLAYER HOPPING

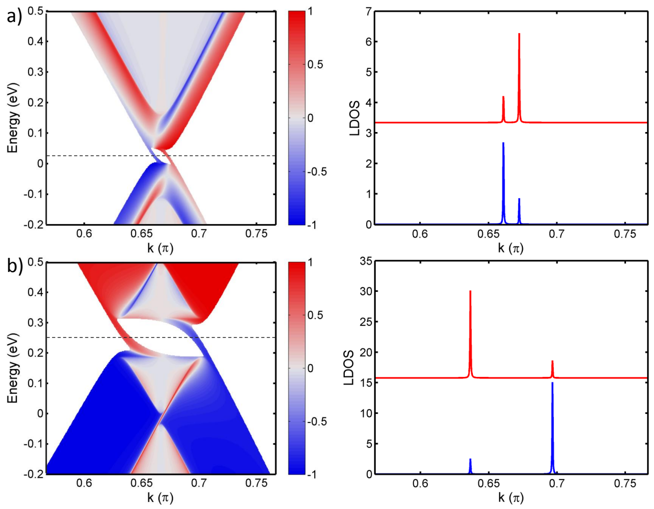

FIG. S1. (Color online) Topological modes of bilayer graphene with a stacking domain wall for smaller γ_1 ($= 0.05\gamma_1$) interlayer coupling, indicative of large screening between layers. Layer resolved LDOS as a function of wave vector and energy (left) and LDOS values at Fermi level (right) for gate voltage values: $V = 0.05$ eV (a) and $V = 0.5$ eV (b).

SI2. PERIODIC STACKING DOMAIN WALLS

The length W of the bilayer superlattice unit cell is measured by the number of 8-atom units, as marked in Fig. S2 (a). The system is made periodic perpendicular to DW by including two stacking boundaries with reverse effect: one to change from AB to BA (marked as DW) and another one to change from BA to AB stacking ($\overline{\mathrm{DW}}$), as shown in Fig. S2 (a). Strain is accumulated in the bonds of the top layer in one case and of the bottom layer in the second [1].

Figure S2 (b) presents the band structure of the superlattice for a large unit cell, $W = 40$. The dispersion relation is plotted for the wave vector k along the stacking boundary direction, in order to compare to the k-resolved LDOS presented in Fig. 1 of the manuscript. The spectra are in fact rather similar, with the obvious difference that in the superlattice there are now four topological modes (two pairs that cross each other) due to the existence of two stacking domain walls in the unit cell, one pair with positive velocity and the other with opposite slope. The dispersion of these modes is clearly seen in the zoom. Decreasing the superlattice spatial period W, as in Fig. S2 (c,d), the linear portions of the topological bands become larger. So the peculiar shape of topological bands as presented in the single domain wall of Fig. 1 of the manuscript, or in the zoom for a large superlattice, as in S2(b), is due to the repulsion from the rest of the valence and conduction bands. Note that these modes disappear for $V = 0$ or if the interlayer hopping is eliminated, because either way the gap is closed, as mentioned before. A common procedure to study the occurrence of gapless modes is to consider the gate potential as a perturbation.

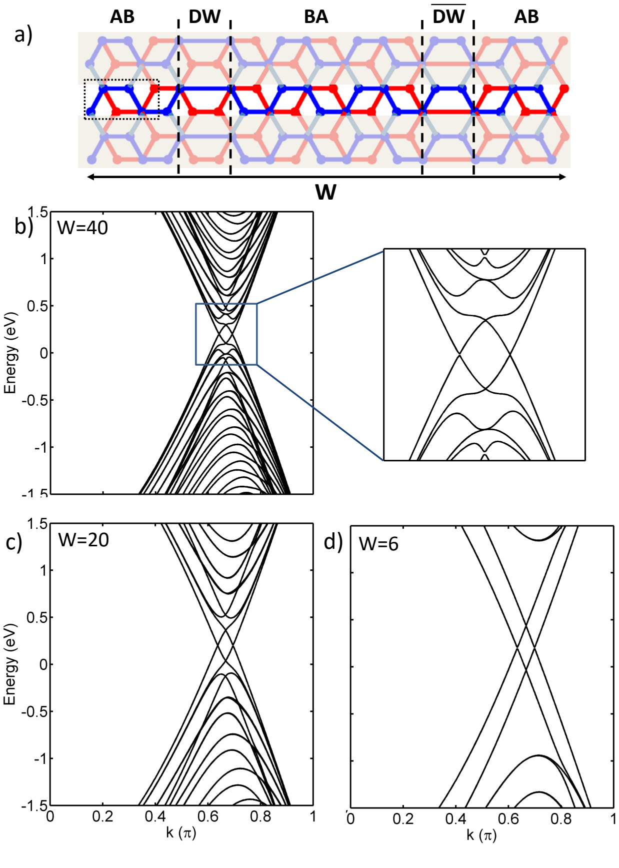

FIG. S2. (Color online) (a) Schematic geometry of the unit cell for a bilayer superlattice with two stacking domain walls. AB, BA and domain walls are delimited with dashed lines. The 8-atom cell used as unit of length is marked with a dotted rectangle. (b) Band structure near the K point along the k zigzag direction of a $W = 40$ BS-DW. A zoom of the topological modes is shown at the right panel. Band structures for (c) $W = 20$ and (d) $W = 6$ BS-DW in the same k direction as (b). In all cases the gate voltage is set to $V = 0.4$ eV.

It is noteworthy that for $V > 0$ and $\gamma_1 = 0$ the bands of the constituent layers cross near the Fermi level. For nonzero γ_1 the crossing bands interact and split yielding the energy gap in the case of pristine bilayer. However, when a stacking domain wall is imposed, two bands still persist in the gap. The treatment of γ_1 as a perturbation allows to recognize the bands of single graphene layers that give rise to the presence of topological bands in the energy gap.

---

[1] In fact, the boundaries consist of a different bonding of the atoms of the bottom and the top layer, without actually modifying the numerical value of the hopping.
